# *tert*-Butyl 4-cyano­phenyl carbonate

**DOI:** 10.1107/S1600536810038602

**Published:** 2010-09-30

**Authors:** Malcolm J. Applewhite

**Affiliations:** a Department of Chemistry and Polymer Science, Stellenbosch University, Private Bag X1, Matieland 7602, South Africa

## Abstract

The title compound, C_12_H_13_NO_3_, was prepared by reacting one equivalent of di-*tert*-butyl dicarbonate with 4-cyano­phenol. Herringbone crystal packing is observed and there are no significant inter­molecular inter­actions.

## Related literature

For a similar packing arrangement in related structures, see: Girard *et al.* (2005 ▶); Nagata *et al.* (2008 ▶). For reference structural data, see: Allen *et al.* (1987 ▶).
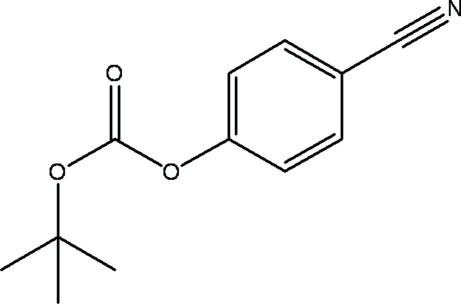


## Experimental

### 

#### Crystal data

C_12_H_13_NO_3_
*M* *_r_* = 219.23
Monoclinic, 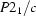

*a* = 5.7347 (7) Å
*b* = 14.3237 (16) Å
*c* = 13.7727 (16) Å
β = 101.110 (1)°
*V* = 1110.1 (2) Å^3^
*Z* = 4
Mo *K*α radiation
μ = 0.10 mm^−1^
*T* = 296 K
0.45 × 0.25 × 0.12 mm

#### Data collection

Bruker APEX CCD area-detector diffractometer
Absorption correction: multi-scan (*SADABS*; Bruker, 2009 ▶) *T*_min_ = 0.959, *T*_max_ = 0.989
6536 measured reflections
2427 independent reflections
2100 reflections with *I* > 2σ(*I*)
*R*_int_ = 0.017

#### Refinement

*R*[*F* ^2^ > 2σ(*F* ^2^)] = 0.034
*wR*(*F* ^2^) = 0.091
*S* = 1.05
2427 reflections
148 parameters
H-atom parameters constrained
Δρ_max_ = 0.31 e Å^−3^
Δρ_min_ = −0.23 e Å^−3^

### 

Data collection: *APEX2* (Bruker, 2009 ▶); cell refinement: *SAINT* (Bruker, 2009 ▶); data reduction: *SAINT*; program(s) used to solve structure: *SHELXS97* (Sheldrick, 2008 ▶); program(s) used to refine structure: *SHELXL97* (Sheldrick, 2008 ▶); molecular graphics: *X-SEED* (Barbour, 2001 ▶; Atwood & Barbour, 2003 ▶); software used to prepare material for publication: *X-SEED* (Barbour, 2001 ▶; Atwood & Barbour, 2003 ▶).

## Supplementary Material

Crystal structure: contains datablocks I, global. DOI: 10.1107/S1600536810038602/bh2315sup1.cif

Structure factors: contains datablocks I. DOI: 10.1107/S1600536810038602/bh2315Isup2.hkl

Additional supplementary materials:  crystallographic information; 3D view; checkCIF report

## Figures and Tables

**Fig. 1.**
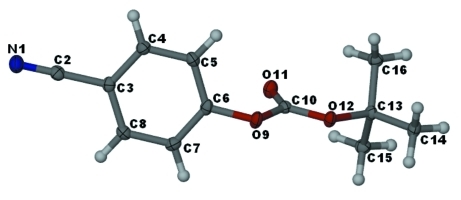
The molecular structure of the title compound showing the 50% probability ellipsoids for non-H atoms.

**Fig. 2.**
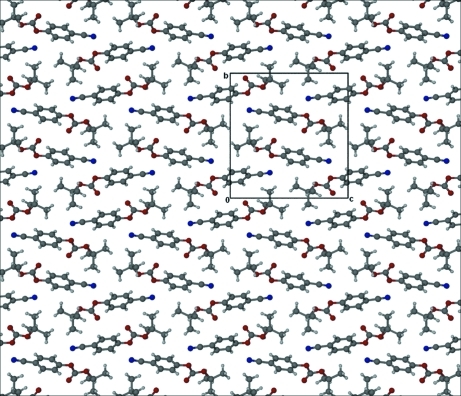
The crystal packing viewed down the a axis which shows the herring-bone type packing and the two oppositely oriented molecules in each turn.

## supplementary crystallographic information

### Comment

In the title molecule, all bond lengths after refinement are within the normal values as previously collected by Allen *et al.* (1987).

The two-dimensional herring-bone type packing along the *b* and *c* axis observed has two molecules oriented in opposite directions in each turn. Such an arrangement is similar to that described by Girard *et al.* (2005) and Nagata *et al.* (2008) for related organics.

### Experimental

Di-*tert*-butyl dicarbonate (279 mg, 1.28 mmol) was added to 4-cyano phenol in 5 ml of dichloromethane. 4-Dimethylamino pyridine (5.20 mg, 0.043 mmol) as a catalyst and triethylamine (0.263 ml, 1.87 mmol) as a base were also added. The solution was stirred at room temperature for six hours.

The reaction was then worked-up using 20 ml of H_2_O and 10 ml of 2*M* HCl which was repeated. The solution was then dried over MgSO_4_ after which the solvent was reduced. The resultant white solid was then dried thoroughly under vacuum.

### Refinement

Structure solution and refinement were performed using the *SHELX97* suite of programs (Sheldrick, 2008). The H atoms were placed in calculated positions, using and refined using a riding model, with C—H bond lengths fixed to 0.93 (aromatic CH) or 0.96 Å (methyl CH_3_).

### Crystal data

**Table:**

C_12_H_13_NO_3_	*F*(000) = 464
*M**_r_* = 219.23	*D*_x_ = 1.312 Mg m^−^^3^
Monoclinic, *P*2_1_/*c*	Mo *K*α radiation, λ = 0.71073 Å
Hall symbol: -P 2ybc	Cell parameters from 3143 reflections
*a* = 5.7347 (7) Å	θ = 2.8–27.5°
*b* = 14.3237 (16) Å	µ = 0.10 mm^−^^1^
*c* = 13.7727 (16) Å	*T* = 296 K
β = 101.110 (1)°	Plate, colourless
*V* = 1110.1 (2) Å^3^	0.45 × 0.25 × 0.12 mm
*Z* = 4	

### Data collection

**Table:**

Bruker APEX CCD area-detector diffractometer	2427 independent reflections
Radiation source: fine-focus sealed tube	2100 reflections with *I* > 2σ(*I*)
graphite	*R*_int_ = 0.017
ω scans	θ_max_ = 27.9°, θ_min_ = 2.1°
Absorption correction: multi-scan (*SADABS*; Bruker, 2009)	*h* = −7→7
*T*_min_ = 0.959, *T*_max_ = 0.989	*k* = −18→14
6536 measured reflections	*l* = −17→15

### Refinement

**Table:**

Refinement on *F*^2^	Primary atom site location: structure-invariant direct methods
Least-squares matrix: full	Secondary atom site location: difference Fourier map
*R*[*F*^2^ > 2σ(*F*^2^)] = 0.034	Hydrogen site location: inferred from neighbouring sites
*wR*(*F*^2^) = 0.091	H-atom parameters constrained
*S* = 1.05	*w* = 1/[σ^2^(*F*_o_^2^) + (0.0448*P*)^2^ + 0.3462*P*] where *P* = (*F*_o_^2^ + 2*F*_c_^2^)/3
2427 reflections	(Δ/σ)_max_ < 0.001
148 parameters	Δρ_max_ = 0.31 e Å^−^^3^
0 restraints	Δρ_min_ = −0.23 e Å^−^^3^
0 constraints	

### Fractional atomic coordinates and isotropic or equivalent isotropic displacement parameters (Å^2^)

**Table:**

	*x*	*y*	*z*	*U*_iso_*/*U*_eq_	
N1	0.33241 (18)	0.70351 (7)	0.16270 (7)	0.0241 (2)	
C2	0.4174 (2)	0.69212 (8)	0.24446 (8)	0.0182 (2)	
C3	0.52163 (19)	0.67936 (8)	0.34783 (8)	0.0163 (2)	
C4	0.74719 (19)	0.71664 (8)	0.38553 (8)	0.0180 (2)	
H4	0.8322	0.7469	0.3438	0.022*	
C5	0.84213 (19)	0.70792 (8)	0.48552 (8)	0.0187 (2)	
H5	0.9911	0.7325	0.5117	0.022*	
C6	0.7118 (2)	0.66212 (8)	0.54586 (8)	0.0170 (2)	
C7	0.4902 (2)	0.62391 (8)	0.50998 (8)	0.0187 (2)	
H7	0.4072	0.5931	0.5521	0.022*	
C8	0.3944 (2)	0.63258 (8)	0.40994 (8)	0.0182 (2)	
H8	0.2459	0.6073	0.3843	0.022*	
O9	0.79952 (14)	0.65945 (6)	0.64836 (6)	0.0199 (2)	
C10	0.97513 (19)	0.59643 (8)	0.68019 (8)	0.0159 (2)	
O11	1.04663 (14)	0.53999 (6)	0.62887 (6)	0.0211 (2)	
O12	1.04148 (14)	0.61088 (6)	0.77662 (5)	0.01731 (19)	
C13	1.22277 (19)	0.54939 (8)	0.83729 (8)	0.0161 (2)	
C14	1.2463 (2)	0.59433 (9)	0.93865 (8)	0.0210 (3)	
H14A	1.3021	0.6573	0.9359	0.032*	
H14B	1.3575	0.5594	0.9860	0.032*	
H14C	1.0943	0.5947	0.9581	0.032*	
C15	1.1255 (2)	0.45053 (8)	0.83660 (9)	0.0203 (2)	
H15A	0.9751	0.4516	0.8574	0.030*	
H15B	1.2350	0.4123	0.8811	0.030*	
H15C	1.1050	0.4254	0.7709	0.030*	
C16	1.45548 (19)	0.55369 (8)	0.79966 (8)	0.0189 (2)	
H16A	1.4385	0.5204	0.7382	0.028*	
H16B	1.5798	0.5257	0.8475	0.028*	
H16C	1.4948	0.6177	0.7896	0.028*	

### Atomic displacement parameters (Å^2^)

**Table:**

	*U*^11^	*U*^22^	*U*^33^	*U*^12^	*U*^13^	*U*^23^
N1	0.0254 (5)	0.0257 (6)	0.0193 (5)	−0.0037 (4)	−0.0003 (4)	0.0010 (4)
C2	0.0180 (5)	0.0158 (5)	0.0206 (6)	−0.0013 (4)	0.0029 (4)	−0.0008 (4)
C3	0.0179 (5)	0.0151 (5)	0.0150 (5)	0.0023 (4)	0.0012 (4)	−0.0004 (4)
C4	0.0181 (5)	0.0186 (5)	0.0176 (5)	−0.0002 (4)	0.0039 (4)	0.0022 (4)
C5	0.0150 (5)	0.0210 (6)	0.0189 (6)	−0.0004 (4)	0.0007 (4)	0.0001 (4)
C6	0.0187 (5)	0.0184 (5)	0.0135 (5)	0.0058 (4)	0.0019 (4)	0.0013 (4)
C7	0.0201 (5)	0.0181 (6)	0.0191 (6)	0.0015 (4)	0.0070 (4)	0.0025 (4)
C8	0.0158 (5)	0.0175 (5)	0.0207 (6)	−0.0009 (4)	0.0022 (4)	−0.0012 (4)
O9	0.0201 (4)	0.0259 (4)	0.0133 (4)	0.0071 (3)	0.0020 (3)	0.0025 (3)
C10	0.0144 (5)	0.0171 (5)	0.0160 (5)	−0.0008 (4)	0.0029 (4)	0.0025 (4)
O11	0.0240 (4)	0.0221 (4)	0.0167 (4)	0.0043 (3)	0.0026 (3)	−0.0016 (3)
O12	0.0181 (4)	0.0204 (4)	0.0130 (4)	0.0056 (3)	0.0018 (3)	0.0013 (3)
C13	0.0153 (5)	0.0179 (5)	0.0144 (5)	0.0036 (4)	0.0008 (4)	0.0032 (4)
C14	0.0238 (6)	0.0241 (6)	0.0144 (5)	0.0055 (5)	0.0016 (4)	0.0012 (4)
C15	0.0192 (5)	0.0197 (6)	0.0221 (6)	0.0001 (4)	0.0040 (4)	0.0035 (4)
C16	0.0168 (5)	0.0191 (6)	0.0208 (6)	0.0010 (4)	0.0037 (4)	0.0014 (4)

### Geometric parameters (Å, °)

**Table:**

N1—C2	1.1493 (15)	C10—O12	1.3254 (13)
C2—C3	1.4455 (15)	O12—C13	1.4899 (13)
C3—C8	1.3979 (16)	C13—C14	1.5194 (15)
C3—C4	1.4032 (15)	C13—C15	1.5214 (16)
C4—C5	1.3855 (15)	C13—C16	1.5231 (15)
C4—H4	0.9300	C14—H14A	0.9600
C5—C6	1.3850 (16)	C14—H14B	0.9600
C5—H5	0.9300	C14—H14C	0.9600
C6—C7	1.3846 (16)	C15—H15A	0.9600
C6—O9	1.4049 (13)	C15—H15B	0.9600
C7—C8	1.3871 (16)	C15—H15C	0.9600
C7—H7	0.9300	C16—H16A	0.9600
C8—H8	0.9300	C16—H16B	0.9600
O9—C10	1.3605 (13)	C16—H16C	0.9600
C10—O11	1.1971 (14)		
			
N1—C2—C3	178.86 (13)	O12—C13—C14	101.18 (8)
C8—C3—C4	120.57 (10)	O12—C13—C15	109.34 (9)
C8—C3—C2	119.85 (10)	C14—C13—C15	111.57 (9)
C4—C3—C2	119.55 (10)	O12—C13—C16	110.35 (9)
C5—C4—C3	119.42 (10)	C14—C13—C16	111.52 (9)
C5—C4—H4	120.3	C15—C13—C16	112.32 (9)
C3—C4—H4	120.3	C13—C14—H14A	109.5
C6—C5—C4	119.01 (10)	C13—C14—H14B	109.5
C6—C5—H5	120.5	H14A—C14—H14B	109.5
C4—C5—H5	120.5	C13—C14—H14C	109.5
C7—C6—C5	122.50 (10)	H14A—C14—H14C	109.5
C7—C6—O9	118.29 (10)	H14B—C14—H14C	109.5
C5—C6—O9	119.07 (10)	C13—C15—H15A	109.5
C6—C7—C8	118.67 (10)	C13—C15—H15B	109.5
C6—C7—H7	120.7	H15A—C15—H15B	109.5
C8—C7—H7	120.7	C13—C15—H15C	109.5
C7—C8—C3	119.82 (10)	H15A—C15—H15C	109.5
C7—C8—H8	120.1	H15B—C15—H15C	109.5
C3—C8—H8	120.1	C13—C16—H16A	109.5
C10—O9—C6	116.18 (9)	C13—C16—H16B	109.5
O11—C10—O12	129.21 (10)	H16A—C16—H16B	109.5
O11—C10—O9	125.05 (10)	C13—C16—H16C	109.5
O12—C10—O9	105.73 (9)	H16A—C16—H16C	109.5
C10—O12—C13	120.28 (8)	H16B—C16—H16C	109.5
			
C8—C3—C4—C5	−0.93 (17)	C7—C6—O9—C10	105.80 (12)
C2—C3—C4—C5	177.14 (10)	C5—C6—O9—C10	−78.37 (13)
C3—C4—C5—C6	0.27 (17)	C6—O9—C10—O11	−5.43 (16)
C4—C5—C6—C7	0.48 (17)	C6—O9—C10—O12	175.49 (9)
C4—C5—C6—O9	−175.16 (10)	O11—C10—O12—C13	−2.85 (17)
C5—C6—C7—C8	−0.55 (17)	O9—C10—O12—C13	176.18 (8)
O9—C6—C7—C8	175.12 (10)	C10—O12—C13—C14	177.84 (9)
C6—C7—C8—C3	−0.12 (17)	C10—O12—C13—C15	−64.35 (12)
C4—C3—C8—C7	0.86 (17)	C10—O12—C13—C16	59.67 (12)
C2—C3—C8—C7	−177.20 (10)		
